# Parent–child attachment and adolescent problematic behavior: the mediating effect of legal emotions

**DOI:** 10.3389/fpsyg.2025.1546895

**Published:** 2025-02-27

**Authors:** He Jianhua, Xu Su, Xu Shuhui

**Affiliations:** ^1^School of Mechanical Engineering, Tongling University, Anhui, China; ^2^Department of Psychology, Wenzhou University, Zhejiang, China

**Keywords:** parent–child attachment, problematic behavior, legal emotions, adolescents, positive legal emotions

## Abstract

**Introduction:**

In criminology, the close relationship between legal emotions and adolescent deviant behavior is well-documented. In psychology, parental attachment is strongly associated with children’s problematic behavior; however, the role of legal emotions in this relationship remains underexamined.

**Methods:**

This study investigates the role of legal emotions in the relationship between adolescent parent–child attachment and problematic behavior. Adolescents completed self-report questionnaires.

**Results:**

Results revealed that both paternal and maternal attachment significantly negatively predicted adolescent problematic behavior, with paternal attachment explaining a larger proportion of the variance. Even after controlling for factors such as gender and grade level, parental attachment significantly negatively predicted adolescent problematic behavior. Legal emotions partially mediated the relationship between parental attachment and problematic behavior.

**Conclusion:**

These findings suggest that fostering positive parent–child relationships and enhancing adolescents’ positive legal emotions may be effective strategies for reducing problematic behaviors among youth.

## 1 Introduction

Adolescence is a period of rapid physical and psychological development. Due to the faster pace of physical growth compared to psychological maturation, adolescents are prone to experiencing internal psychological conflicts, which may lead to a range of problematic behaviors (Lee and Hankin, 2009). Problematic behavior refers to the maladaptive responses and negative emotional or behavioral reactions that arise when individuals struggle to adapt effectively to changes in their environment, including challenges within family, school, and societal contexts (Lee and Hankin, 2009). It primarily includes externalizing problems such as aggression and rule-breaking, as well as internalizing problems such as depression and anxiety (Gao et al., 2019; Achenbach, 1991). Adolescent problematic behavior affects their mental health and has a significant negative impact on their development (Fosco et al., 2024; Dubowitz et al., 2020; Yap and Jorm, 2015). It may also escalate into more serious criminal behavior. Relevant data indicate that the prevalence of depression among adolescents has reached 11.3%, the incidence of problematic behavior has been as high as 39%, and the occurrence rate of school bullying has reached 53.5% (Cao et al., 2024; Li, 2025). Therefore, investigating the antecedent variables of adolescent problematic behavior and the relationships among these variables is of critical importance.

### 1.1 Parent–child attachment and problematic behavior

Bowlby (1970) developed attachment theory, which primarily focuses on the relationship between infants and their primary caregivers (Bowlby, 1970). Attachment is an emotional bond in which an individual seeks proximity to a target attachment figure (e.g., caregiver) and, through interactions within the caregiving environment, constructs a system composed of one or more individuals who serve as a secure base (Ainsworth et al., 1978). Bowlby (1970) defined attachment relationships as an adaptive function related to the life cycle, rather than being limited to a specific developmental period. In middle childhood, attachment relationships remain highly important, as children continue to need and rely on attachment figures throughout childhood and adolescence (Bowlby, 1989). Various psychodynamic theories suggest that the nature and quality of a child’s first interpersonal relationships are crucial for mental health and personality development (Bowlby, 2010). Therefore, parent–child attachment is a lasting emotional bond formed between children and their primary caregivers (parents) (Ainsworth, 1989).

When children engage with parents who are responsive and supportive, they tend to build self-confidence and demonstrate resilience in coping with stress from adverse events (Kullik and Petermann, 2013). In contrast, interactions with parents who show limited sensitivity and responsiveness are associated with a higher likelihood of maladaptive behaviors and difficulties in interpersonal relationships (Wei et al., 2005). Attachment theory posits that secure attachment to caregivers creates an “internal working model,” which regulates an individual’s cognition, emotions, and behaviors, ultimately influencing various developmental outcomes (Carlo et al., 2012). Parents serve not only as powerful primary caregivers but also as important attachment figures for adolescents, particularly in times of stress (Hay and Ashman, 2003).

Compared to childhood, the probability of problematic behaviors in adolescence, such as misconduct and academic adjustment issues, has significantly increased (Cai and Zhou, 2006). Parent–child relationships, as an important factor in the family system, are closely related to problematic behaviors in adolescents, such as aggressive behavior and misconduct (Tian and Tian, 2014). General Strain Theory posits that parental verbal and behavioral aggression serves as a source of strain for adolescents, leading to the development of aggressive behavior (Agnew, 1992). Related studies also show that lower quality of attachment to parents is significantly associated with increased symptoms of depression and anxiety in adolescents (Wei et al., 2005). Parent–child attachment is the sole predictor of behavioral problems (Al-Yagon, 2014). Meta-analytic results further support the link between insecure attachment and behavioral problems in middle childhood (Madigan et al., 2013). Lower levels of parent–child attachment are a negative influencing factor for the onset and development of childhood depression (Spruit et al., 2020). The stress-buffering model suggests that parent–child attachment, as a positive factor within the family context, serves as a protective factor in various adverse situations (Cohen and Wills, 1985). Dagan and Sagi-Schwartz (2018) suggested that the influence of paternal attachment and maternal attachment on children differs (Dagan and Sagi-Schwartz, 2018). For example, Richaud de Minzi (2010) found that, compared to paternal attachment, maternal attachment had a more significant impact on feelings of loneliness, while paternal attachment was more predictive of depressive symptoms than maternal attachment (María, 2010). Related studies also indicate that the effects of paternal and maternal attachment on adolescent problematic behaviors vary (Dagan and Sagi-Schwartz, 2018).

Based on this, the study aims to preliminarily examine whether parent–child attachment has a negative predictive effect on adolescent problematic behavior and whether there are differences between paternal attachment and maternal attachment in their influence on problematic behavior. Therefore, the study proposes the following hypotheses:

*H1:* Parent–child attachment significantly negatively predicts adolescent problematic behavior.

*H2:* There are differences in the impact of paternal attachment and maternal attachment on adolescent problematic behavior.

### 1.2 The mediating effect of legal emotions

To better prevent and intervene in adolescent problematic behavior, it is essential to further examine how parent–child relationships influence adolescent problematic behavior. According to emotion-related socialization theory, emotional parenting is a direct method of emotional socialization and occurs through parents’ responses to emotions or discussions about emotions between parents and children (Eisenberg and Spinrad, 1998). In the process of emotion socialization, the ability to perceive, understand, and regulate emotions can be directly shaped, presenting both risks and protective factors for children’s problematic behavior (Johnson et al., 2017).

Legal emotions are one component of emotion socialization and a key indicator of legal socialization. Legal emotions, as a subcategory of emotions, refer to an individual’s complex response to legal stimuli, such as the spirit of the rule of law, the current legal system, and its operation. These emotions reflect a psychological phenomenon related to the individual’s desires and needs. Legal emotions are classified into positive and negative legal emotions based on their valence structure. Positive legal emotions refer to the subjective experience of pleasure that arises when the spirit of the rule of law or the current legal system and its functioning align with an individual’s expectations or needs regarding the law. These emotions reflect an individual’s identification with the law and represent an emotional state that the state seeks to promote and cultivate through education. Negative legal emotions, in contrast, are adverse psychological experiences caused by the spirit of the rule of law or the current legal system and its functioning, such as disappointment, disdain, and aversion toward the law and its associated phenomena (Xu et al., 2024).

Theoretically, emotional parenting and parent–child attachment, as both the process and outcome of parent–child interaction, have been viewed as a reciprocal and bidirectional relationship (Morris et al., 2010). Emotion socialization theory indicates that a positive and warm family atmosphere effectively promotes the development of positive emotions and emotional experiences in adolescents while inhibiting negative emotional experiences (Eisenberg et al., 2001; Morris et al., 2010). Legal emotions, as a component of both legal socialization and emotion socialization, are naturally influenced by parents (McGloin and O’Neill Shermer, 2009; Cavanagh and Cauffman, 2015). They can also be regarded as one of the manifestations of emotional parenting.

Related studies identify an interconnection between parent–child attachment and problematic behavior, as well as between emotional parenting and problematic behavior. When a cascading model is used to examine the joint relationship between the two parental factors and problematic behavior, it is observed that parent–child attachment more stably predicts emotional parenting than emotional parenting predicts parent–child attachment. Furthermore, emotional parenting plays a key mediating role in the influence of parent–child attachment on later problematic behavior (Wang et al., 2021). However, the above studies examine the mediating role of emotional parenting in the relationship between parent–child attachment and problematic behavior, and it remains unclear whether their conclusions are applicable to legal emotions. Additionally, related studies find that maternal attachment significantly negatively predicts negative legal emotions, and negative legal emotions significantly positively predict aggressive behavior in college students (Xu et al., 2024). These findings, however, were based on college student samples, and it remains to be further tested whether these conclusions apply to middle school students in China.

Therefore, this study proposes the following hypothesis:

*H3:* Legal emotions mediate the relationship between parent–child attachment and adolescent problematic behavior. Specifically, both paternal attachment and maternal attachment significantly positively predict positive legal emotions, which in turn significantly negatively predict adolescent problematic behavior; both paternal attachment and maternal attachment significantly negatively predict negative legal emotions, which in turn significantly positively predict adolescent problematic behavior.

**Figure 1:**
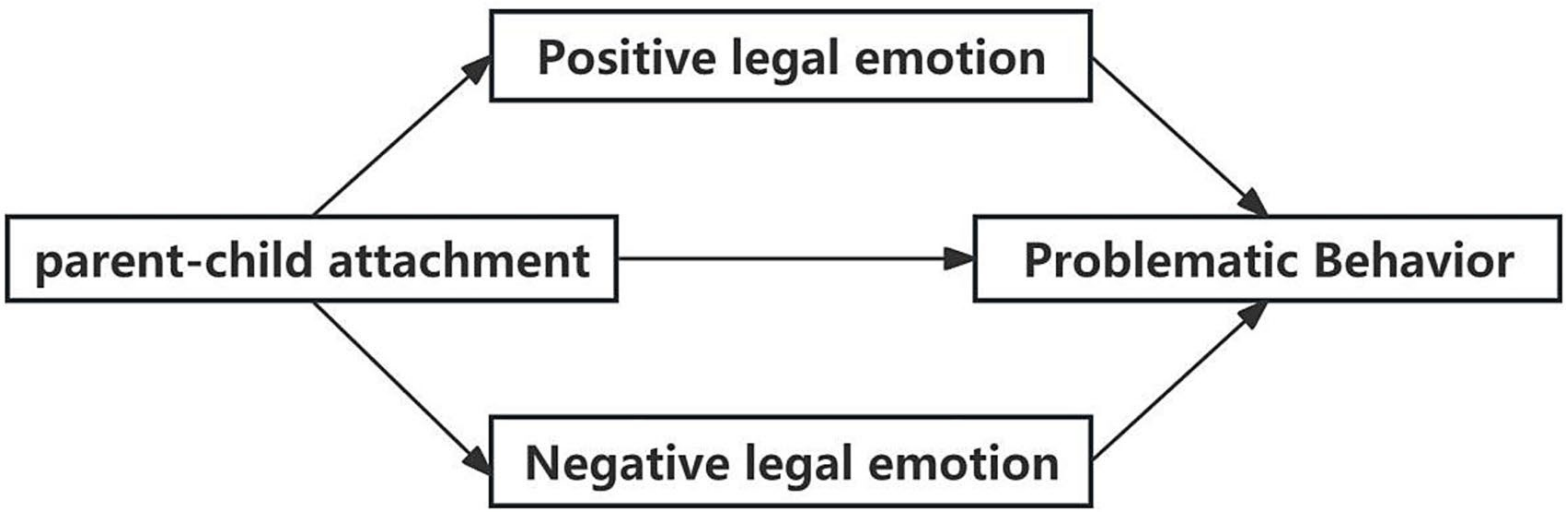
Theoretical hypothesis model.

In summary, this study aims to construct a mediation model (see Figure 1) to examine the impact of parent–child attachment on problematic behavior in Chinese adolescents, as well as the mediating role of legal emotions. The following hypotheses are proposed:

*H1:* Both paternal attachment and maternal attachment in Chinese adolescents can negatively predict problematic behavior.

*H2:* There are differences in the impact of paternal attachment and maternal attachment on adolescent problematic behavior.

*H3:* Positive and negative legal emotions mediate the relationship between parent–child attachment and problematic behavior. Specifically, both paternal attachment and maternal attachment significantly positively predict positive legal emotions, which in turn significantly negatively predict adolescent problematic behavior; both paternal attachment and maternal attachment significantly negatively predict negative legal emotions, which in turn significantly positively predict adolescent problematic behavior.

## 2 Methods

### 2.1 Transparency and openness

This study adheres to the Transparency and Openness Promotion (TOP) guidelines, aiming to ensure transparency and reproducibility throughout the research process. All data and materials utilized in this study are available upon request from the corresponding author, with reasonable justification. Detailed information regarding the study’s compliance with ethical review is provided in the supplementary materials. To support replication, we have clearly described the statistical methods used, and access to the complete dataset can be requested from the corresponding author. These efforts align with the journal’s submission guidelines and promote open science practices. This study was not preregistered.

### 2.2 Participants

This study employed a convenience sampling method to recruit 432 middle school students from a secondary school in Wenzhou, Zhejiang Province, China, to participate in the survey. The survey was administered by psychology students as experimenters, with the participants tested in groups by class. The sample consisted of 228 male and 204 female students, aged 12 to 15 years, with a mean age of 13.37 years (SD = 0.51 years). Prior to the survey, both parents and students signed informed consent forms. Additionally, this study was approved by the ethics review committee of the institution of one of the authors.

#### 2.2.1 Parent–child attachment

The Parent Attachment Questionnaire, a revised version of the Parent and Peer Attachment Scale developed by Raja et al. (1992), was used in this study. The scale consists of three dimensions: trust, communication, and alienation, with a total of 10 items. Sample items include, “I tell my father about the problems and difficulties I encounter,” and “My father respects my feelings.” Participants rated each item on a 5-point Likert scale, where 1 represents “never” and 5 represents “always.” Some items were reverse-scored to maintain balance. Higher total scores indicated stronger attachment, while lower scores indicated weaker attachment. The internal consistency coefficients (Cronbach’s *α*) for paternal attachment and maternal attachment in this study were 0.88 and 0.87, respectively.

#### 2.2.2 Problematic behavior

This questionnaire was a revised version of the Problematic Behavior Self-Report Questionnaire for Students, developed by Professor Cui Lixia from Capital Normal University (Cui and Zheng, 2005). The original questionnaire consisted of 60 items, divided into six factors: academic maladjustment, aggressive behavior, rule-breaking behavior, withdrawal, neuroticism, and test anxiety. This study primarily investigates the following problematic behaviors among middle school students: academic maladjustment (e.g., not paying attention in class, failing to complete assignments on time). Academic maladjustment refers to non-cognitive negative behaviors that result in poor academic performance, including neglecting homework, procrastination, lack of concentration, and inattentiveness. Aggressive behavior (e.g., hitting others) and rule-breaking behavior (e.g., cheating on exams, not following the rules) are considered externalizing behavioral problems within the category of problematic behaviors. Therefore, 31 items from the original questionnaire were selected for this study. The items were rated using a 4-point Likert scale, where 1 represented “never” and 4 represented “always.” Higher scores indicated more problematic behavior. The overall internal consistency coefficient (Cronbach’s *α*) for the three dimensions in this study was 0.92.

#### 2.2.3 Legal emotions

The Adolescent Legal Emotions Scale was developed by Xu and Yan (2022). The scale consists of two subscales: Positive Legal Emotions and Negative Legal Emotions. The scale contains 43 items in total, with 20 items for the Positive Legal Emotions subscale (e.g., “I am very interested in court trials,” “I believe that the law can make our lives better”) and 23 items for the Negative Legal Emotions subscale (e.g., “I dislike the coercive nature of the law,” “I have doubts about the fairness of the People’s Procuratorate”). The scale uses a 5-point Likert rating system, ranging from “Strongly Disagree” to “Strongly Agree.” Higher scores indicate a higher level of either positive or negative legal emotions in the participants. The Cronbach’s α coefficients for the two subscales were 0.957 and 0.935, respectively. In this study, the internal consistency coefficients (Cronbach’s α) for Positive Legal Emotions and Negative Legal Emotions were 0.95 and 0.97, respectively.

#### 2.2.4 Covariate

Previous research has shown that parental education level plays a significant role in the psychological development of children and adolescents (Liu et al., 2016). Additionally, studies have demonstrated a close relationship between adolescent problem behaviors and age (Liu et al., 2016). Attachment theory suggests that, with age, an individual’s attachment to their parents tends to decrease (Brown and Larson, 2009). Therefore, apart from gender, parental education level and age were included as covariates in this study to control for their potential effects on adolescent problem behaviors and attachment.

### 2.3 Statistical analysis

SPSS 22.0 was used to conduct common method bias tests, correlation analysis, regression analysis, and other statistical procedures. The Bootstrap method proposed by Hayes was employed to test for mediation effects (Preacher and Hayes, 2004).

## 3 Results

### 3.1 Common method bias test

Common method bias was tested using Harman’s single-factor test (Zhou and Long, 2004). Seventeen factors had eigenvalues greater than 1, and the variance explained by the first factor was 24.90%, which is below 40%. Therefore, no significant common method bias was found in the data of this study.

### 3.2 Descriptive statistics and correlation coefficients for all variables

Descriptive statistics and correlation analysis were conducted for paternal attachment, maternal attachment, legal emotions, and problematic behaviors, with the results presented in Table 1. Paternal attachment was significantly positively correlated with both maternal attachment and positive legal emotions and significantly negatively correlated with negative legal emotions. Additionally, paternal attachment was significantly negatively correlated with problematic behaviors. Maternal attachment showed a significant positive correlation with positive legal emotions, a significant negative correlation with negative legal emotions, and a significant negative correlation with problematic behaviors. Positive legal emotions were significantly negatively correlated with negative legal emotions and significantly negatively correlated with problematic behaviors. Negative legal emotions were significantly positively correlated with problematic behaviors. An independent samples t-test was conducted to examine gender differences in each variable. The results revealed significant gender differences in paternal attachment, maternal attachment, and positive legal emotions, with males scoring significantly higher than females on both paternal attachment and positive legal emotions. Therefore, gender was controlled as a covariate in subsequent analyses. Correlational and descriptive statistics for each variable are presented in Table 1.

**Table 1:** Descriptive statistics and correlation analysis of research variables (*n* = 432).

Variable	1	2	3	4	5	6	7
1. Age	1						
2. Gender	0.02	1					
3. PA	−0.03	0.11^*^	1				
4. MA	−0.04	0.07	0.69^***^	1			
5. PLE	−0.04	0.10^*^	0.28^***^	0.30^***^	1		
6. NLE	0.06	0.04	−0.27^***^	−0.32^***^	−0.50^***^	1	
7. PB	0.02	0.01	−0.41^***^	−0.40^***^	−0.25^***^	0.30^***^	1
*M ± SD*			34.71 ± 8.91	37.71 ± 8.38	86.62 ± 14.38	34.41 ± 17.28	50.95 ± 13.06
*M ± SD(M)*			35.64 ± 9.08	38.33 ± 8.26	87.91 ± 14.97	34.99 ± 19.64	51.02 ± 14.04
*M ± SD(F)*			33.67 ± 8.63	37.01 ± 8.47	85.19 ± 13.56	33.76 ± 14.25	50.86 ± 11.90
*t*			2.29^*^	1.63	1.98^*^	0.73	0.12

Gender(M, Male; F, Female); PA, paternal attachment; MA, maternal attachment; PLE, positive legal emotions; NLE, negative legal emotions; PB, problematic behavior. *p < 0.05; **p < 0.01; ***p < 0.001.

### 3.3 Regression analysis of paternal attachment and maternal attachment on adolescent problematic behaviors

The stepwise multiple regression results indicated that both paternal attachment and maternal attachment significantly predicted adolescent problematic behaviors. The multiple correlation coefficient between the two predictors and problematic behaviors was 0.475, and the coefficient of determination (*R*^2^) was 0.226. The *F*-value for the overall regression model was 53.125 (*p* = 0.000 < 0.05), indicating that the two predictors together explained 22.6% of the variance in problematic behaviors. Specifically, paternal attachment accounted for 19.6% of the variance, while maternal attachment explained 3%. The standardized regression coefficients (*β*) for both predictors were negative, indicating that both predictors had a negative effect on problematic behaviors. Details are presented in Table 2.

**Table 2:** Summary of stepwise multiple regression analysis of paternal attachment and maternal attachment on problematic behaviors.

Sequence of input variables	*R*	*R^2^*	Increase (△*R^2^*)	*F*	△*F*	*B*	*β*
(Constant)						77.686	
paternal attachment	0.411	0.169	0.169	85.989^***^	88.816^***^	−0.360	−4.06
maternal attachment	0.446	0.199	0.030	52.419^***^	15.818^***^	−0.376	−3.97

*p < 0.05; **p < 0.01; ***p < 0.001.

The mediation effect was tested using the PROCESS plugin, with a 95% confidence interval and 5,000 bootstrap samples for the bias-corrected percentile bootstrap method. Model 4 was selected, controlling for gender, age, and parents’ educational level. Paternal attachment/maternal attachment was set as the independent variable, problematic behaviors as the dependent variable, and positive legal emotions as the mediator for mediation effect analysis (see Table 3). The results showed that the direct effect of paternal attachment on problematic behaviors was significant. Paternal attachment significantly positively predicted positive legal emotions. When both paternal attachment and positive legal emotions were used to predict problematic behaviors, the negative predictive effect of positive legal emotions on problematic behaviors remained significant. These findings suggest that positive legal emotions partially mediate the relationship between paternal attachment and problematic behaviors. See Table 4.

**Table 3:** Mediation effect model testing with positive legal emotions as the mediator and paternal pttachment as the independent variable.

Variable	Problematic behavior		Positive legal emotion		Problematic behavior	
	*β*	*t*	*β*	*t*	*β*	*t*
Constant	0.005	0.004	0.642	0.529	0.087	0.075
Paternal attachment	−0.401	−8.869^***^	0.247	5.206^***^	−369	−7.983^***^
Positive legal emotion					−0.127	−2.744^**^
Gender	0.114	1.285	0.121	1.300	0.130	1.466
Age	−0.018	−0.211	−0.033	−0.365	−0.023	−0.261
Father’s level of education	0.068	1.374	−0.097	−1.861	0.056	1.130
Mother’s level of education	0.004	0.082	−0.007	−0.142	0.003	0.064
*R^2^*	0.177		0.096		0.192	
*F*	17.932^***^		8.816^***^		16.432^***^	

**p < 0.01; ***p < 0.001.

**Table 4:** Decomposition of direct, indirect, and total effects with positive legal emotions as the mediator and paternal attachment as the independent variable.

	Effect size	BootSE	BootLLCL	BootULCL
Total effect	−0.401	0.045	−0.490	−0.312
Direct effect	−0.369	0.046	−0.460	−0.279
Indirect effect	−0.031	0.015	−0.064	−0.006

The direct effect of maternal attachment on problematic behaviors was significant. Maternal attachment significantly positively predicted positive legal emotions. When both maternal attachment and positive legal emotions were used to predict problematic behaviors, the negative predictive effect of positive legal emotions on problematic behaviors remained significant. These findings indicate that positive legal emotions partially mediated the relationship between maternal attachment and problematic behaviors. See Tables 5, 6.

**Table 5:** Mediation effect model test with positive legal emotion as the mediator and maternal attachment as the independent variable.

Variable	Problematic Behavior		Positive legal Emotion		Problematic Behavior	
	*β*	*t*	*β*	*t*	*β*	*t*
Constant	0.128	0.111	0.431	0.362	0.186	0.162
Maternal attachment	−0.391	−8.474^***^	0.263	5.515^***^	−0.356	−7.507^***^
Positive legal emotion					−0.135	−2.878^**^
Gender	0.074	0.832	0.141	1.532	0.093	1.051
Age	−0.020	−0.225	−0.023	−0.260	−0.023	−0.263
Father’s level of education	0.073	1.459	−0.098	−1.895	0.060	1.201
Mother’s level of education	−0.037	−0.770	0.024	0.474	−0.034	−0.710
*R^2^*	0.162		0.099		0.178	
*F*	16.282^***^		9.277^***^		15.183^***^	

**p < 0.01; ***p < 0.001.

**Table 6:** Decomposition of direct, indirect, and total effects with positive legal emotion as the mediator and maternal attachment as the independent variable.

	Effect size	BootSE	BootLLCL	BootULCL
Total effect	−0.391	0.046	−0.482	−0.301
Direct effect	−0.356	0.047	−0.449	−0.263
Indirect effect	−0.035	0.017	−0.071	−0.007

Using the same method, the mediating effect of negative legal emotions between paternal attachment, maternal attachment, and problematic behaviors was tested. The results showed that paternal attachment had a significant direct effect on problematic behaviors. Paternal attachment significantly negatively predicted negative legal emotions. When both paternal attachment and negative legal emotions were used to predict problematic behaviors, the positive predictive effect of negative legal emotions on problematic behaviors remained significant. These findings indicate that negative legal emotions partially mediated the relationship between paternal attachment and problematic behaviors. See Tables 7, 8.

**Table 7:** Mediation effect model testing with negative legal emotion as the mediator and paternal attachment as the independent variable.

Variable	Problematic Behavior		Negative legal emotion		Problematic Behavior	
	*β*	*t*	*β*	*t*	*β*	*t*
Constant	0.005	0.004	−1.512	−1.224	0.308	0.272
Paternal Attachment	−0.401	−8.869^***^	−0.276	−5.715^***^	−0.346	−7.528^***^
Negative legal emotion					0.201	4.470^***^
Gender	0.114	1.285	0.145	1.520	0.085	0.978
Age	−0.018	−0.211	0.105	1.130	−0.039	−0.462
Father’s level of education	0.068	1.374	−0.035	−0.656	0.075	1.547
Mother’s level of education	0.004	0.082	0.052	1.027	−0.007	−0.141
*R^2^*	0.177		0.084		0.215	
*F*	17.932^***^		7.619^***^		18.953^***^	

***p < 0.001.

**Table 8:** Decomposition of direct, indirect, and total effects with negative legal emotion as the mediator and paternal attachment as the independent variable.

	Effect size	BootSE	BootLLCL	BootULCL
Total effect	−0.401	0.045	−0.490	−0.312
Direct effect	−0.346	0.046	−0.436	−0.255
Indirect effect	−0.055	0.018	−0.097	−0.024

Maternal attachment had a significant direct effect on problematic behaviors. Maternal attachment significantly negatively predicted negative legal emotions. When both maternal attachment and negative legal emotions were used to predict problematic behaviors, the positive predictive effect of negative legal emotions on problematic behaviors remained significant. These findings indicate that negative legal emotions partially mediated the relationship between maternal attachment and problematic behaviors. See Tables 9, 10.

**Table 9:** Mediation effect model test with negative legal emotion as the mediator and maternal attachment as the independent variable.

Variable	Problematic behavior		Negative legal emotion		Problematic behavior	
	*β*	*t*	*β*	*t*	*β*	*t*
Constant	0.128	0.111	−1.284	−1.073	0.378	0.334
Maternal attachment	−0.391	−8.474^***^	−0.329	−6.869^***^	−0.327	−6.851^***^
Negative legal emotion					0.195	4.236^***^
Gender	0.074	0.832	0.126	1.365	0.050	0.566
Age	−0.020	−0.225	0.096	1.061	−0.038	−0.448
Father’s level of education	0.073	1.459	−0.036	−0.695	0.080	1.631
Mother’s level of education	−0.037	−0.770	0.013	0.268	−0.040	−0.841
*R^2^*	0.162		0.110		0.196	
*F*	16.282^***^		10.392^***^		17.105^***^	

***p < 0.001.

**Table 10:** Decomposition of direct, indirect, and total effects in the mediation model with negative legal emotion as the mediator and maternal attachment as the independent variable.

	Effect size	BootSE	BootLLCL	BootULCL
Total effect	−0.391	0.046	−0.482	−0.301
Direct effect	−0.327	0.048	−0.421	−0.233
Indirect effect	−0.064	0.022	−0.111	−0.027

## 4 Discussion

This study examined the impact of parent–child attachment on problematic behavior among Chinese adolescents. The results showed that both paternal and maternal attachment significantly negatively predicted problematic behavior, with paternal attachment demonstrating a higher predictive rate than maternal attachment. Furthermore, legal emotions mediated the relationship between both forms of attachment and problematic behavior. These findings support the theoretical hypotheses.

After controlling for the influence of demographic variables on problematic behavior, the regression analysis conducted with paternal attachment and maternal attachment as predictor variables revealed that both paternal and maternal attachment significantly and negatively predicted problematic behavior. Moreover, the explanatory power of paternal attachment for problematic behavior was greater than that of maternal attachment. Parent–child attachment is a proximal factor in children’s behavior and is closely related to problematic behavior in children (Rehder et al., 2021). Attachment theory posits that attachment relationships develop through the interactions and exchanges between children and their parents (Ma and Huebner, 2008). When parents provide appropriate feedback and meet the attachment needs of children in a timely manner, this facilitates the formation of attachment between the child and caregiver, leading to the development of positive internal working models and reducing the occurrence of problematic behaviors (Kerns and Brumariu, 2014). Research shows that poor parent–child attachment further leads to developmental and adaptive issues in children (Wu et al., 2022). Secure attachment to parents is associated with fewer problematic behaviors (Dykas and Cassidy, 2011).

This study found that paternal attachment predicted adolescent problematic behaviors more strongly than maternal attachment, which is consistent with previous research (Pan et al., 2016). The reason for this may lie in the different roles parents play in an individual’s development (Li, 2020). Mothers primarily care for their children’s daily lives and provide emotional support, while fathers symbolize discipline and authority (Deng et al., 2013; Lewis and Lamb, 2003). Therefore, in the context of adolescent problematic behaviors such as misconduct and aggression, the influence of paternal attachment was stronger.

This study further indicated that both positive and negative legal emotions partially mediated the relationship between parent–child attachment and problematic behavior, suggesting that an individual’s parent–child attachment influences problematic behavior through legal emotions. According to the attachment theory proposed by John Bowlby, the attachment patterns formed in early parent–child relationships have a significant impact on an individual’s psychological and behavioral development. A positive parent–child relationship helps children form secure attachments, and this sense of security fosters the development of positive emotions (Bartholomew and Horowitz, 1991). Based on this reasoning, it can also be inferred that such security promotes the development of positive legal emotions.

The theory of authoritative relationships in legal socialization posits that the personal and substitute experiences of young people with both legal and non-legal authority figures have a significant impact on their legal socialization (Tyler and Trinkner, 2018). The transmission of legal values begins in early childhood and continues throughout life through individuals’ interactions with the legal system (Trinkner and Tyler, 2016). The internalization of legal values provides a foundational basis for individuals to judge the legitimacy of laws and legal institutions (Bottoms and Tankebe, 2012). Parents play a particularly prominent role in the early stages of legal socialization, transmitting attitudes and beliefs to their children and influencing their understanding of the law and legal authority, thereby shaping their value systems. Parents also pass on their own legal orientations to their children, directly affecting their attitudes toward the legitimacy of the criminal justice system and its agents (Cavanagh and Cauffman, 2015). As authority figures, parents shape how children view authority and the legitimacy of the law by setting an example of fairness and appropriately exercising authority (Trinkner and Cohn, 2014). Research has shown that positive parent–child communication helps children form positive values (Yu, 2009). Based on this, it can be inferred that positive parent–child attachment contributes to the formation of positive legal emotions. Therefore, the more positive the parent–child attachment, the more it contributes to the development of positive legal emotions in adolescents and inhibits negative legal emotions.

The Internal Working Model in attachment theory provides an effective framework to explain why individuals with secure attachment are better able to interact harmoniously with others, as they possess stable and positive beliefs and expectations about themselves and interpersonal relationships (Bretherton and Munholland, 2008). Therefore, positive parent–child attachment can positively predict an individual’s interpersonal relationships, reducing the likelihood of problematic behaviors such as aggression or bullying. In contrast, negative parent–child attachment, due to the absence of a well-formed internal model for harmonious interactions with others, is more likely to result in tense interpersonal relationships, such as aggression or bullying. Law can be seen as an institutionalized social contract designed to maintain social order and interpersonal harmony. A strong parent–child attachment reflects a friendly model of interpersonal relationships. During an individual’s development, this internal working model is generalized to interactions with others, including their relationship with the law, which regulates social relationships. Therefore, positive parent–child attachment predicts positive legal emotions, which represent an acknowledgment of the importance of maintaining social order and harmonious relationships. As a result, positive legal emotions negatively predict conflict-driven interpersonal behaviors such as bullying, aggression, and rule-breaking. In contrast, low levels of parent–child attachment represent a conflictual model of interpersonal relationships. This model reflects disdain and disregard for laws that maintain harmonious relationships and undermines the authority and binding power of the law. Hence, negative parent–child attachment positively predicts negative legal emotions, and negative legal emotions positively predict adolescent problematic behaviors.

This research finding can also be interpreted within the framework of social learning theory (Bandura and Cliffs, 1986). In the context of parent–child interactions, the behaviors, attitudes, and values exhibited by parents have a direct impact on the behavioral patterns of children. When parents display positive legal emotions, such as respect for laws and rules, children are more likely to model these behaviors, thereby developing similar legal emotions and attitudes. This process fosters a habit of adhering to rules and laws, ultimately reducing the likelihood of problematic behaviors. Conversely, if the parent–child relationship is characterized by aggression or emotional detachment, children are more prone to replicate these negative interaction patterns in their dealings with others and with society at large. This may lead to a rejection of the laws that underpin social order, fostering negative emotions toward legal authority and, consequently, an increase in behaviors such as aggression, rule-breaking, and other forms of deviant conduct. Therefore, parent–child attachment can directly influence adolescents’ problematic behaviors and indirectly affect these behaviors through the mediation of legal emotions.

The legal emotions that can be deeply ingrained in the hearts of citizens must inevitably arise from a profound legal culture within a nation. China’s legal culture is rooted in Confucianism as its inner spirit, encapsulated in the principle of “incorporating rites into law.” Since this study uses Chinese adolescents as participants, the interpretation of the results should consider the influence of social and cultural factors. The theory of emotional social structure posits that emotional experiences are rooted in rational social context relationships, and emotions are acquired within the socio-cultural system (Fu, 2016). Legal emotions are a type of emotion formed during the emotional socialization process. The current laws of a country are, in fact, a confirmation and commitment to the mainstream culture of society. As individuals in a given social period share a certain legal system, their cognition of this legal system becomes the foundation of legal emotions. This cognition, in turn, is influenced by interpersonal interactions and reflects relationships between individuals. In other words, legal emotions are primarily formed through interpersonal relationships and can also influence these relationships. The fact that they are shaped within interpersonal interactions while simultaneously shaping these interactions demonstrates the social control power of legal emotions (Xu, 2020).

This perspective can also be explained from a cultural standpoint, highlighting the inhibitory effect of parent–child attachment and positive legal emotions on adolescent problematic behavior observed in this study. Western research on legal emotions is rooted in individualistic cultures, emphasizing the impact of the procedural justice model on individual legal socialization (Jonathan-Zamir et al., 2015). Western studies often focus on legal cynicism, defined as an emotional state of contempt and pessimism toward the law, and its impact on individuals’ subsequent behavior (Sampson and Bartusch, 1998). Therefore, although adolescents’ legal emotions in Western countries are also influenced by various relationships, there are cultural differences in the foundational basis of social relationships. Chinese adolescents tend to recognize and comply with authority, while adolescents in Western countries place more emphasis on “negotiation” within relationships and the “legitimacy” of authority.

## 5 Limitations and future directions

This study has several limitations. First, the self-report method was used, which may introduce bias. Future research could adopt a multi-method and multi-rater approach to assess adolescents’ problematic behaviors, legal emotions, and parent–child attachment.

Second, this study only examined the role of legal emotions in the relationship between parent–child attachment and adolescents’ problematic behaviors. Previous studies have shown that mothers’ adult attachment directly and indirectly influences children’s behavioral problems through maternal emotional socialization (Bao and Kato, 2023). In other words, parents’ adult attachment may serve as a model for the emotional expression and regulation patterns of the next generation, potentially affecting children’s behavior (Parrigon et al., 2015; Cowan et al., 2009). Related research indicates that parents’ secure attachment in their family of origin is negatively correlated with children’s externalizing problems, while insecure attachment is positively correlated with both internalizing and externalizing problems (Roskam et al., 2011; Kohlhoff et al., 2023). Furthermore, a longitudinal study showed that caregivers’ adult attachment had a greater impact on children’s behavioral problems than caregivers’ psychopathology, such as depression (Zajac and Kobak, 2009). Future research could incorporate the parental adult attachment variable into the model of this study for further exploration. This can also be examined through the integrated theoretical model of problematic behavior, which considers the influence of both the personality system and the situational system on adolescent problematic behavior (Jessor et al., 2003). For example, future studies could include variables such as psychological resilience, family functioning, peer relationships in the classroom, and legal emotions into the research model. This approach aims to reveal the mechanisms among various protective factors and provide more effective strategies for preventing and treating adolescent problematic behavior.

Third, the participants in this study were adolescents aged 13 to 15. The impact of parent–child attachment and legal emotions on adolescents may vary with individual age and years of education. Specifically, from early to late adolescence, the influence of parents on individuals gradually decreases (Chan et al., 2022). With exposure to more legal education courses, adolescents develop a deeper understanding of the law, which, in turn, influences their legal emotions. Therefore, it is uncertain whether the conclusions of this study can be generalized to older adolescents. Additionally, cross-sectional designs often fail to accurately capture the longitudinal relationships underlying mediation processes. Mediation inherently involves causal processes that unfold over time, and cross-sectional designs do not account for prior levels of mediators and outcome variables, potentially leading to significant biases in estimating direct and indirect effects (Maxwell and Cole, 2007; Cole David and Maxwell, 2003). Future studies should adopt longitudinal designs to more accurately examine mediation effects and capture the temporal relationships between variables, reducing biases in causal inferences.

Fourth, parent–child attachment can directly influence adolescent problematic behavior and also exert an indirect effect through legal emotions. Therefore, the prevention and intervention of adolescent problematic behavior can be approached through multiple strategies. Family counseling can enhance parent–child relationships, while behavior management workshops can strengthen these connections. Social and emotional development programs, such as cultivating positive legal emotions through case-based teaching or mock trial activities, can also be incorporated. These strategies collectively contribute to the prevention and treatment of adolescent problematic behavior.

## Data Availability

The raw data supporting the conclusions of this article will be made available by the authors, without undue reservation.
